# OP06 The Specifics Of Public Opposition To Negative Reimbursement Decisions In Health Care: A Systematic Review

**DOI:** 10.1017/S0266462325100652

**Published:** 2025-12-29

**Authors:** Vivian Reckers-droog, Saskia Knies, Philipa Mos, Werner Brouwer

## Abstract

**Introduction:**

Decisions to not reimburse or discontinue reimbursement of health technologies from public funding are considered increasingly necessary. However, such decisions remain politically sensitive and often provoke public opposition. Such opposition may be considerable and puts pressure on decision-makers to revoke or revise a decision; however, the specifics of this opposition remain largely unclear. Our aim was to obtain insight into these specifics by mapping the scientific literature on this topic.

**Methods:**

We performed a systematic review following PRISMA guidelines. The electronic search was performed in Embase and using the Google Scholar, Google, and Startpage search engines and supplemented with a hand search in 2021. Both the electronic searches and the hand search were updated in 2022 and 2024.

**Results:**

Based on 81 articles, we developed a framework of 27 categories grouped under the ‘who, what, when, where, and why’ of public opposition to negative reimbursement decisions. Our findings indicated that patient representatives, physicians, and citizens are the primary actors opposing such decisions—often influenced by industry and politicians. These actors typically challenge the outcomes and arguments underlying decisions, driven by unrealistically high expectations of technology effectiveness and the assumption that decision-makers prioritize cost containment above all else. Additionally, our findings indicate increasing distrust among these actors toward decision-makers and evidence-based processes, leaving them vulnerable to commercial exploitation and misinformation.

**Conclusions:**

Understanding the dynamics between actors is crucial for understanding public opposition to negative reimbursement decisions in health care. Aligning decision-making processes with public values, addressing misconceptions, and countering misinformation may enhance the legitimacy of reimbursement decisions. Further research should explore strategies to mitigate distrust and foster evidence-based engagement among actors to ensure more informed and equitable healthcare decision-making.

